# Curing the incurable: A case of refractory vasospastic angina

**DOI:** 10.1016/j.amsu.2021.102869

**Published:** 2021-09-15

**Authors:** David Song, Mohamed Abdelghffar, Tasur Seen, Andrew Kim, XiongBin Lin, Saad Ahmad, Talal Almas, Julie Kanevsky

**Affiliations:** a Department of Internal Medicine Icahn School of Medicine at Mount Sinai - Elmhurst Hospital Center, Elmhurst, NY, USA; b New York Institute of Technology College of Osteopathic Medicine, Westbury, NY, USA; c Royal College of Surgeons in Ireland, Dublin, Ireland

**Keywords:** Vasospastic angina, Coronary artery disease, Serotonin receptor inhibitor

## Abstract

Vasospastic angina (VSA) is the spasm of coronary arteries causing transient myocardial ischemia. VSA is commonly managed with antispasmodic medications including calcium-channel blockers (CCB) and nitrates. When vasospasm is refractory to conventional medications, unconventional treatment modalities may be used for symptomatic relief (Tandon et al., 2019 Feb) [1]. There are several mediators of vasospasm, with serotonin playing a major role. Serotonin is a product of platelet aggregation and has multiple effects on the endothelium, which forms the basis of an unconventional treatment modality that may be used for symptomatic relief of VSA. In this case, a 44 year old female with a history of coronary artery disease (CAD) status post coronary artery bypass graft (CABG) with recent drug-eluting stent (DES) placement was admitted for shortness of breath and chest pain, found to have a positive stress echocardiogram (Echo), and had unremarkable coronary angiography. Given persistent symptoms while on optimal medical therapy and with negative coronary angiography, the diagnosis of refractory VSA was made. Patient was started on a serotonin receptor blocker with improvement of her symptoms.

## Highlights

• Vasospastic angina (VSA) occur through smooth muscle hyperactivity and serotonin mediates coronary vasospasm.
• Refractory VSA not responding to standard treatment, the use of serotonin antagonists should be considered.
• Further trials of the serotonin receptor families and the properties of individual antagonist may have clinical benefit.

## 1 Introduction

VSA is a variant form of angina pectoris, where angina occurs at rest, with transient electrocardiographic changes and preserved exercise capacity. Coronary vasospasm is a heterogeneous phenomenon that can occur in patients with or without coronary atherosclerosis. It can be focal or diffuse, and can affect epicardial or microvascular coronary arteries [2]. The clinical picture of VSA is similar to obstructive CAD except that angina is non-exertional. It has a high incidence in individuals less than 50 years of age and there is an association with other vasospastic disorders like Raynaud's disease [3]. The gold standard for diagnosis uses invasive coronary angiography or directly imaging coronary spasm using acetylcholine, ergonovine or methylergonovine as provocative stimuli. Treatment consists of lifestyle changes aimed at removal of provoking stimuli, avoidance of vasospastic agents and the use of pharmacotherapy such as CCB, nitrates, statins, aspirin, alpha1-adrenergic receptor antagonists, or serotonin receptor antagonist [2]. This work has been reported in accordance with SCARE [4].

## 2 Definition

VSA was originally described by Prinzmetal et al. as “a variant type of angina in which the pain comes on with the subject at rest or during ordinary activity during the day or night with transient ST-segment elevation on electrocardiogram and preserved exercise capacity. The episodes almost always terminate spontaneously, but if it persists, it may lead to death”. However, the definition of VSA has undergone several adaptations over the years. The *Coronary Vasomotion Disorders International Study Group (COVADIS)* has recently proposed guidelines for diagnostic criteria of VSA (Table 1). Recently, the European Society of Cardiology (ESC) 2018 guidelines on ST-elevation myocardial infarction emphasized the importance of myocardial infarction (MI) with non-obstructive coronary arteries, encouraging the treating physician to investigate the underlying cause such as VSA [5].

**Table 1:** Coronary Artery Vasospastic Disorders Summit diagnostic criteria for vasospastic angina [10].

1. *1)Nitrate-responsive angina*—during an episode, with at least one of the following a. Rest angina—especially between night and early morning b. Marked diurnal variation in exercise tolerance—reduced in morning c. Hyperventilation can precipitate an episode d. Calcium channel blockers (but not β-blockers) suppress episodes
2. *2)Transient ischemic ECG changes*—during spontaneous episode, including any of the following in at least two contiguous leads: a. ST segment elevation ≥0.1 mV b. ST segment depression ≥0.1 mV c. New negative U waves
3) *Coronary artery spasm*—defined as transient total or subtotal coronary artery occlusion (>90% constriction) with angina and ischemic ECG changes either spontaneously or in response to a provocative stimulus (typically acetylcholine, ergot, or hyperventilation)

## 3 Case presentation

Patient is a 44-year-old female with a past medical history of hypertension, hyperlipidemia, triple vessel CAD s/p CABG (RIMA to LAD, LIMA to OM1, SVG to Ramus) and one DES 8 months prior who presented with complaints of multiple episodes of non-exertional dyspnea and angina for four months. In her most recent episode, she had sudden severe nonradiating substernal chest pain accompanied by shortness of breath worsening on exertion and minimal relief with sublingual nitroglycerin (SLNTG). She had similar episodes twice a week for the previous four months with increasing severity. She was asymptomatic for three years after CABG, but developed anginal symptoms 8 months prior to current admission, was found to have a positive stress echo and coronary angiography revealed a spastic phenomenon of the LIMA with 85% occlusion of ostial LAD, where a DES was placed. Despite the treatment, the patient experienced minimal relief of her symptoms. Diagnosis of VSA was made at that time and SLNTG was started.

Patient's medications included ranolazine 1000mg daily, isosorbide mononitrate 60mg twice daily, metoprolol tartrate 25mg twice daily, diltiazem ER 360mg daily, atorvastatin 80 mg daily, clopidogrel 75mg daily, aspirin 81mg daily and SLNTG as needed for chest pain. Patient had received all the antianginal drugs with no improvement in her symptoms. Her metoprolol dose was limited by bradycardia to the 50s. All medications were continued during hospitalization.

On this admission, the physical exam and vital signs were unremarkable. Her electrocardiogram (ECG) revealed sinus bradycardia with nonspecific ST changes and transthoracic echocardiogram (TTE) showed a normal ejection fraction, but was notable for segmental wall motion abnormality suggestive of ischemia in the left ventricle. Labs including troponins were otherwise unremarkable. Stress echo was concerning for ischemia and given the patient's clinical history, a restenosis of a coronary graft or CAD progression distal to the site of CABG were suspected. However, coronary angiography revealed patent graft vessels, no in-stent restenosis, and a normal left ventricular end diastolic pressure.

**Fig. 1:**
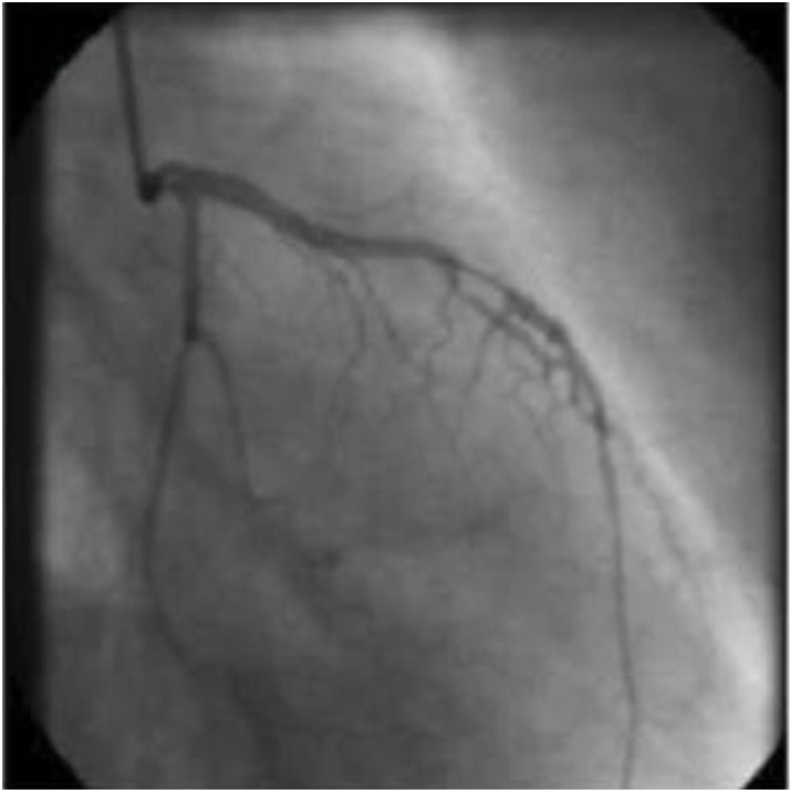
Coronary angiography with ergonovine challenge showing spasm of left anterior descending (LAD).

Considering the patient's young age and severity of the disease, rheumatologic etiologies were considered but rheumatologic workup was negative including antinuclear antibodies, anti-double stranded DNA, anti-histone antibodies, anti-smith antibodies, rheumatoid factor, and antineutrophil cytoplasmic antibody. Given persistence of angina while on optimal medical therapy with patent graft vessels, absence of restenosis, and negative work up for secondary etiologies, a diagnosis of refractory VSA was made. Patient was subsequently started on a 5-hydroxytryptamine antagonist, Cyproheptadine at a dose of 4 mg every 8 hours with marked improvement in anginal symptoms. Coronary angiography with ergonovine challenge was performed, confirming the diagnosis (Fig. 1) and outpatient follow-up after discharge revealed resolution of her symptoms.

## 4 Discussions

VSA contributes to about 2% of all hospital admissions with a clinical picture of unstable angina. There has been an increased incidence of the disease in people less than 50 years of age and there is a male prevalence of 5:1. The data regarding its incidence in the United States is insufficient due to the highly variable presentation of the disease [6]. The disease has a close mimicry with unstable angina, and as a result, it is often missed unless a high index of suspicion is maintained.

VSA can occur due to smooth muscle hyperactivity. Serotonin is an important endocrine mediator of coronary vasospasm and is one of the major products of platelet aggregation [7]. Serotonin can provoke vasospasm by accumulating in platelets. Serotonin binds to 5-hydroxytryptamine receptors on endothelial cells to release vasodilating compounds. When released in healthy endothelium, it causes dilation by removal of platelet aggregation. However, if the endothelium is injured or dysfunctional, vasoconstriction occurs because the endothelium-dependent vasodilation cannot remove the vasoactive substances. The absence of vasodilation produced by healthy endothelium leads to accumulation of platelets. This causes platelet aggregation and vasoconstriction of the arteries [6,7].

Although the initial experience with the use of serotonin receptor antagonists has been disappointing per Willerson JT et al. [8], our patient responded well to cyproheptadine, a 5-hydroxytryptamine antagonist. Similar presentations have been previously reported, where patients were noted to have VSA refractory to nitrates and CCB but were successfully treated with cyproheptadine [9].

In addition to pharmacotherapy, aggressive risk factor modification, such as smoking and alcohol cessation should be recommended. Patients should also be advised to avoid any emotional or physical triggers of their symptoms [1]. If first-line management with CCB and second-line management with nitrates fail after medical optimization, unconventional management should be pursued with the use of serotonin receptor antagonists.

## 5 Conclusion

Although vast advances have been made in the diagnosis and management of VSA, the disease remains elusive due to its myriad of clinical presentations and the varied response to the standard modalities of treatment. Current diagnostic guidelines can help arrive at the correct diagnosis, but clinicians need to maintain a high index of suspicion in young patients with persistent anginal symptoms while on optimal medical regimen and with unremarkable coronary angiography. In cases of refractory VSA not responding to conventional treatment, the use of serotonin antagonists such as cyproheptadine should be considered. Further clinical trials of its effectiveness need to be done, and a better understanding of serotonin receptor families and the unique physiologic properties of individual antagonists may have clinical benefit.

## 6 Limitations

Given the rarity of VSA, there is limited literature available on its treatment, and the optimal dose of cyproheptadine has not been established. The dosage that was used in our patient was from prior report by AD Schecter et al. The effectiveness of the medication was solely evaluated based on the clinical symptoms. Furthermore, the long term sequelae of cyproheptadine and the frequency of follow-up needed after initiation of the drug are not well defined.

## Ethical approval

Obtained.

## Sources of funding

None.

## Author contribution

DS wrote the abstract, case, study concept, design, conclusion;
MA, TS, AK, XL reviewed paper, wrote discussion.
DS, SA, TA, JK final edits.

## Registration of research studies

Name of the registry: NA.

Unique Identifying number or registration ID: NA.

Hyperlink to your specific registration (must be publicly accessible and will be checked): NA.

## Guarantor

Talal Almas

RCSI University of Medicine and Health Sciences.

123 St. Stephen's Green Dublin 2, Ireland.

Talalamas.almas@gmail.com.

+353834212442.

## Consent

Obtained.

## Disclosure

None.

## Declaration of competing interest

None.
